# EEG Connectivity Signatures in Migraine and Tension-Type Headache: A Multimethod ROI-Based Functional Connectivity and Machine-Learning Analysis

**DOI:** 10.3390/jcm15156046

**Published:** 2026-08-04

**Authors:** Zeynep Selcan Sanlı, Ismail Çalıkuşu, Pamir Bastin, Seda Mencekoglu Bastin, Hulya Binokay, Vahide Deniz Yerdelen

**Affiliations:** 1 Department of Neurology, Adana City Training and Research Hospital, 01230 Adana, Türkiye; pamirbastin@gmail.com (P.B.); sedamencekoglu@gmail.com (S.M.B.); yerdelend@gmail.com (V.D.Y.); 2 Biomedical Device Technology Program, Vocational School, Nevşehir Hacı Bektaş Veli University, 50300 Nevsehir, Türkiye; ismailcalikusu@nevsehir.edu.tr; 3 Department of Biostatistics, Çukurova University Faculty of Medicine, 01330 Adana, Türkiye; hbinokay@cu.edu.tr

**Keywords:** migraine, tension-type headache, EEG, functional connectivity, imaginary coherence, wPLI, debiased wPLI, graph theory, machine learning

## Abstract

**Background/Objectives**: Migraine and tension-type headache (TTH) are common primary headache disorders, but their underlying network-level EEG patterns remain incompletely characterized. We examined whether resting-state functional connectivity measured with complementary estimators, predefined sensor-level regions of interest (ROIs), graph features, and machine-learning models could distinguish migraine, TTH, and healthy controls. **Methods**: The study included 150 participants (61 migraine, 47 TTH, and 42 controls). Connectivity was calculated in the delta, theta, alpha, beta, and gamma bands using coherence, imaginary coherence, weighted phase-lag index (wPLI), and debiased wPLI. We evaluated global, regional, topographic, and graph-theoretical features, performed ROC-AUC analyses, and tested classification models with nested cross-validation. To limit the multiple-testing burden, false discovery rate correction was applied to a prespecified, hypothesis-driven ROI set. **Results**: Eight candidate ROI features remained significant after correction. TTH showed the highest gamma temporo-parietal coherence, whereas migraine showed higher gamma temporo-parietal wPLI and debiased wPLI and a higher exploratory migraine probability-like score. Controls had higher delta fronto-temporal and gamma fronto-temporal/temporo-parietal imaginary coherence. Single-feature ROC-AUC values were moderate, and the nested machine-learning models showed only modest classification performance. **Conclusions**: The results point to method-dependent, region-specific sensor-level EEG differences across migraine, TTH, and control groups. They are best viewed as candidate neurophysiological signatures rather than clinically ready biomarkers. External validation, fuller clinical covariate assessment, and source-level analyses are needed before diagnostic use can be considered. Future studies should also examine whether these candidate signatures differ according to aura status and episodic or chronic headache subtype.

## 1. Introduction

Primary headache disorders are a major cause of disability worldwide and often interfere with daily activities, work, and cognitive functioning [1]. Migraine and tension-type headache (TTH) are clinically distinct disorders. Both have been associated with alterations in pain processing and sensory-network function, although the underlying mechanisms and the strength of evidence differ between migraine and TTH [2]. Migraine commonly presents with recurrent attacks, photophobia, phonophobia, nausea, and sensory hypersensitivity. TTH typically presents with bilateral, pressing or tightening pain of mild-to-moderate intensity that is not aggravated by routine physical activity. Stress and pericranial muscle tenderness may contribute in some patients but are not required diagnostic features [3,4,5,6].

Current evidence suggests that primary headaches are not simply episodic pain syndromes. Their clinical expression reflects an interaction among central nervous system mechanisms, peripheral musculoskeletal factors, psychological stress, sleep, and lifestyle. In TTH, craniocervical muscle dysfunction, myofascial trigger points, central sensitization, coping style, and physical activity may contribute to heterogeneity and chronification [6,7,8,9]. Migraine likewise involves abnormal cortical excitability, altered sensory gain, trigeminovascular activation, and changes in distributed pain-related networks [3,4,5,10,11,12,13]. This overlap, together with disorder-specific features, creates a clear need for objective neurophysiological measures that can be interpreted alongside the clinical examination.

Sleep is one clinically important example of this interaction. Poor sleep quality, insomnia symptoms, irregular sleep schedules, and circadian disturbance are frequently reported in both migraine and TTH. Shared mechanisms involving hypothalamic and brainstem pathways, dopaminergic signaling, neuroinflammation, central sensitization, and arousal regulation may influence both headache burden and chronification [14]. EEG may therefore be informative, but its findings should be interpreted together with variables such as sleep, attack frequency, pain severity, and disease duration.

EEG is non-invasive, widely available, and well suited to studying oscillatory activity and network dynamics because of its high temporal resolution [3,4]. Even so, results across headache studies remain inconsistent. Differences in patient selection, electrode density, recording conditions, reference choice, preprocessing, artifact handling, and connectivity metrics can all change the observed pattern [3,4,5]. Resting-state EEG and MEG studies increasingly support persistent network alterations in migraine [3,4,5,10,11,12,13], whereas direct three-group comparisons of migraine, TTH, and healthy controls are still uncommon.

The four connectivity measures used here describe related but non-identical aspects of coupling. Coherence captures linear synchronization but is strongly influenced by zero-lag activity and volume conduction. Imaginary coherence, wPLI, and debiased wPLI place greater weight on non-zero phase-lag relationships and are less sensitive—though not immune—to these effects. Comparing them within the same dataset can therefore reveal whether a group difference is driven mainly by shared zero-lag activity, delayed interactions, or both [10,15].

Recent methodological work has further emphasized the importance of reliable preprocessing and connectivity-estimation strategies in resting-state EEG network analysis. Çelik and Aydın investigated resting-state EEG connectivity using directed transfer function, imaginary coherence, and wPLI together with ICA- and ASR-based preprocessing pipelines, and reported that connectivity reliability and graph-theoretical findings can vary substantially according to the selected estimator and artifact-reduction strategy [16]. Their findings showed that graph-theoretical EEG connectivity can capture alterations in large-scale network segregation, integration, modularity, and resilience, while also underlining that sensor-level EEG connectivity should be interpreted cautiously because graph nodes represent scalp electrodes rather than direct cortical sources [16]. This methodological perspective supports the use of multiple connectivity estimators, graph-theoretical metrics, and cautious sensor-level interpretation in the present study.

Previous EEG- and network-based studies have reported altered coherence, microstate dynamics, oscillatory activity, and phase-lag-sensitive connectivity in migraine [10,11,12,13,15]. In parallel, emerging neuroimaging, fMRI, and MEG evidence suggests that TTH is associated with abnormal connectivity and network topology involving frontal, temporal, thalamic, somatomotor, insular, visual-related, and pain-modulation systems [7,8,9]. The recent clinical and rehabilitation literature also emphasizes the role of cervical dysfunction, myofascial mechanisms, spinal manipulation, dry needling, and multimodal physiotherapy approaches in headache management, further supporting the concept that headache disorders involve both central and peripheral network-related mechanisms [17].

In addition to conventional EEG analysis, recent studies increasingly use source-localization and network-based approaches such as LORETA, isolated effective coherence, and directional connectivity to investigate neural communication at the source level [18]. These approaches provide stronger anatomical interpretability than sensor-level analysis and highlight the importance of source-level confirmation in future EEG connectivity studies. However, sensor-level EEG remains clinically valuable because it is accessible, practical, and suitable for exploratory network-level biomarker discovery, particularly when interpreted cautiously and supported by phase-lag-sensitive metrics.

Machine learning has become increasingly common in EEG-based neurological classification, including migraine research [19]. Reported performance, however, is highly dependent on sample structure, feature engineering, validation design, and protection against information leakage. For that reason, we used nested cross-validation, restricted all preprocessing and feature handling to the training folds, and emphasized balanced metrics rather than accuracy alone. The models were intended as an exploratory test of multivariable signal, not as diagnostic tools.

Few studies have combined several EEG connectivity estimators, predefined ROIs, graph measures, ROC analysis, and leakage-conscious machine learning in a single migraine–TTH–control cohort. Bringing these components together allows a more direct test of whether the disorders differ in specific method–frequency–region combinations rather than through one uniform connectivity abnormality.

The primary objective was to compare resting-state EEG connectivity and graph-theoretical network organization among migraine, TTH, and healthy controls. A secondary objective was to assess the discriminative value of selected features using ROC-AUC analysis and nested cross-validated machine learning. Because the full feature space was large, we also performed a restricted, hypothesis-driven ROI analysis with false discovery rate control. This design was intended to improve interpretability while preserving the exploratory scope of the study.

Previous studies have generally examined a single connectivity estimator, a single headache disorder, or source- and sensor-level findings in separate analytical frameworks. Direct three-group comparisons of migraine, TTH, and healthy controls that integrate conventional coherence, phase-lag-sensitive estimators, predefined sensor-level ROIs, graph measures, and leakage-conscious nested machine learning remain scarce. The present study brings these complementary approaches together in one clinically characterized cohort and explicitly distinguishes zero-lag-sensitive from lag-sensitive connectivity patterns. We hypothesized that (i) migraine and TTH would show distinct frequency- and method-dependent sensor-level connectivity profiles; (ii) temporo-parietal and fronto-temporal candidate ROIs would show the clearest group differences; and (iii) these features would provide modest, rather than clinically definitive, discrimination in internally validated machine-learning models.

## 2. Materials and Methods

### 2.1. Study Design and Participants

This prospective observational clinical study investigated whether resting-state EEG-derived functional connectivity, region-based connectivity profiles, graph-theoretical network measures, and candidate biomarker scores could differentiate patients with migraine, patients with tension-type headache (TTH), and healthy controls. Participants were recruited from the Neurology Outpatient Clinic of Adana City Training and Research Hospital between December 2025 and May 2026. Clinical diagnoses of migraine and TTH were established by an experienced neurologist according to the International Classification of Headache Disorders, third edition (ICHD-3), criteria. For the purposes of this study, interictal recording was operationally defined as EEG acquisition outside an active headache attack and at least 24 h after headache resolution. All EEG recordings were also performed at least 24 h after the last use of acute analgesic medication. None of the patients were receiving preventive headache treatment at the time of EEG acquisition. These criteria were applied to reduce the potential effects of recent headache attacks, the early postdromal period, and acute medication use on EEG connectivity measures.

Resting-state EEG recordings were acquired using a Cadwell EEG system (Cadwell Industries, Inc., Kennewick, WA, USA). Statistical analyses were performed using IBM SPSS Statistics version 26.0 (IBM Corp., Armonk, NY, USA), while EEG signal processing, connectivity analysis, graph-theoretical calculations, and machine-learning analyses were conducted using MATLAB R2023b (The MathWorks, Inc., Natick, MA, USA).

The study was designed as a sensor-level exploratory EEG connectivity study. Therefore, the main analytical focus was on scalp-level connectivity patterns rather than direct cortical source-level interactions. The overall workflow consisted of participant recruitment, clinical classification, resting-state EEG acquisition, preprocessing, frequency-band-specific connectivity estimation, extraction of global and regional connectivity features, graph-theoretical analysis, statistical testing, and exploratory machine-learning-based classification.

### 2.2. Inclusion and Exclusion Criteria

Participants aged 18 years or older with a clinical diagnosis of migraine or TTH and technically adequate EEG recordings were included in the patient groups. Healthy controls were included if they had no history of primary headache disorder, neurological disease, or psychiatric disease.

Exclusion criteria were major systemic disease, neurological disease other than primary headache, epilepsy, psychiatric disorders, pregnancy, breastfeeding, malignancy, inflammatory disease, regular antiepileptic or antidepressant use, corticosteroid or anticoagulant treatment, prophylactic headache treatment, and excessive analgesic use. EEG recordings with insufficient technical quality, excessive artifacts, or inadequate numbers of clean epochs after preprocessing were excluded from the final connectivity analysis. Patients receiving preventive treatment for migraine or TTH were excluded. Occasional use of acute analgesic medication was permitted, provided that the EEG recording was performed at least 24 h after the most recent dose. Participants whose EEG was recorded during a headache attack or within 24 h of the most recent attack were not included in the analysis.

### 2.3. Ethical Approval and Informed Consent

The study was approved by the Scientific Research Ethics Committee of Adana City Training and Research Hospital, Adana Provincial Health Directorate, Türkiye (meeting no. 11, decision no. 416, approval date: 6 March 2025). All procedures were conducted in accordance with the Declaration of Helsinki. Written informed consent was obtained from all participants before data collection. All EEG and clinical data were anonymized before analysis, and no personally identifiable information was used during statistical analysis or reporting.

### 2.4. EEG Recording and Preprocessing

Resting-state EEG recordings were acquired under standardized clinical conditions in a quiet room. Participants were seated comfortably and instructed to remain relaxed during the recording. EEG data were recorded at a sampling rate of 256 Hz. The original routine clinical EEG recordings included 32 channels. For the present connectivity analyses, 21 standard scalp EEG channels were retained to provide a consistent montage across participants: Fp1, Fp2, T1, F7, F3, Fz, F4, F8, T2, T3, C3, Cz, C4, T4, T5, P3, Pz, P4, T6, O1, and O2.

Signals were band-pass filtered between 0.5 and 45 Hz, and a 50 Hz notch filter was applied to reduce power-line noise. The continuous EEG signals were segmented into non-overlapping 4 s epochs. Artifact-contaminated epochs were rejected using amplitude-based criteria, peak-to-peak activity, excessive frontal activity, and robust variance-based criteria. Channels and epochs with excessive noise were excluded before connectivity estimation. Only technically adequate EEG segments were retained for participant-level connectivity analysis.

**Figure 1:**
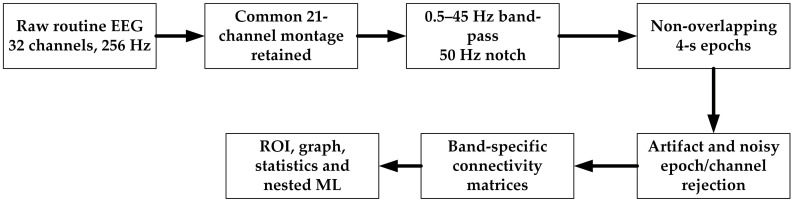
Preprocessing and analytical workflow from raw routine EEG acquisition to connectivity, ROI, graph-theoretical, statistical, and nested machine-learning analyses.

The preprocessing sequence was applied in the same order for all participants: montage harmonization to the 21 retained electrodes; band-pass and notch filtering; segmentation into non-overlapping 4 s epochs; automated rejection based on amplitude, peak-to-peak activity, excessive frontal activity, and robust variance; exclusion of persistently noisy channels/epochs; confirmation that an adequate number of clean epochs remained; computation of epoch-level connectivity matrices; and averaging to participant-level matrices. All quality-control decisions preceded group-level statistical testing. Because the source recordings were routine clinical EEGs, uniform ICA decomposition and source reconstruction could not be applied retrospectively. Figure 1 summarizes the complete processing pathway from raw EEG to statistical and machine-learning analysis.

Because the available EEG recordings were obtained in a routine clinical setting, advanced high-density EEG procedures, source reconstruction, and uniform independent component analysis-based artifact correction were not applied. This limitation was considered when interpreting the results. Therefore, all connectivity findings are reported as sensor-level network patterns rather than direct anatomical or cortical source-level connectivity.

### 2.5. Connectivity Measures and Frequency Bands

Connectivity analyses were performed separately in the following conventional EEG frequency bands: delta, 0.5–4 Hz; theta, 4–8 Hz; alpha, 8–13 Hz; beta, 13–30 Hz; and gamma, 30–45 Hz.

Four complementary connectivity measures were calculated for each participant, frequency band, and electrode pair: coherence, imaginary coherence, weighted phase-lag index (wPLI), and debiased wPLI. Coherence was used to estimate conventional linear synchronization between electrode pairs. Imaginary coherence, wPLI, and debiased wPLI were included to emphasize non-zero phase-lag relationships and to reduce, although not completely eliminate, the influence of zero-lag coupling and volume conduction.

Connectivity matrices were calculated for each clean 4 s epoch and then averaged across retained epochs to obtain participant-level connectivity matrices for each connectivity method and frequency band. For each participant, diagonal self-connections were excluded from subsequent analyses. The resulting matrices represented scalp-level functional connectivity estimates among the retained 21 EEG channels.

### 2.6. Global, Regional, and Graph-Theoretical Features

For each participant, the upper triangular part of each connectivity matrix, excluding diagonal elements, was used to calculate global connectivity features. These included global mean connectivity, standard deviation of connectivity values, maximum connectivity, and weak, moderate, and high connection counts.

Region-based analyses were performed using predefined sensor-level regions of interest (ROIs). ROI-level connectivity values were calculated by averaging the connectivity values of predefined electrode pairs within each ROI. The predefined ROI electrode-pair definitions are provided in Table A2 to allow replication of the regional analyses.

ROI selection rationale and timing: The fronto-temporal, fronto-parietal, temporo-parietal, fronto-central, centro-parietal, and posterior pair sets were chosen because prior EEG, MEG, and neuroimaging studies have implicated frontal executive/pain-modulatory, temporal sensory, parietal multisensory-attentional, and posterior visual-processing systems in migraine and TTH [3,4,5,7,8,9,10,11,12,13,15]. The restricted inferential ROI set placed particular emphasis on fronto-temporal and temporo-parietal connections because these regions recur in reports of altered sensory integration, cortical excitability, pain modulation, and network topology. The electrode-pair definitions and the restricted hypothesis set were finalized before group-level significance testing and before inspection of corrected *p* values; no ROI was added or removed in response to the final statistical results. These labels describe scalp-electrode-pair groupings and must not be interpreted as anatomical source-localized cortical regions.

Weighted graph-theoretical metrics were calculated from sparse weighted networks after thresholding. The graph metrics included mean degree/strength, clustering coefficient, global efficiency, local efficiency, and betweenness centrality. These metrics were used to characterize network segregation, integration, and centrality properties at the sensor level. Because graph metrics can be affected by thresholding strategy and sensor montage, the graph-theoretical findings were interpreted as exploratory network descriptors rather than definitive neurophysiological biomarkers.

Region-based analyses were performed using predefined sensor-level ROIs. ROI-level connectivity values were calculated by averaging the connectivity values of predefined electrode pairs within each ROI. The predefined ROI electrode-pair definitions used for regional EEG connectivity analysis are provided in Appendix A.

### 2.7. Clinical Metadata and Covariate Sensitivity Analysis

Available clinical metadata were extracted from the clinical records and summarized by diagnostic group. These variables included age, sex, headache history, monthly headache days, visual analog scale score, and headache subtype. Migraine was classified as episodic or chronic according to ICHD-3 criteria; chronic migraine required headache on at least 15 days per month for more than 3 months, with migraine features on at least 8 days per month. TTH was classified as episodic or chronic according to ICHD-3 monthly headache-day and duration criteria. Aura status was recorded as migraine with or without aura when this information was available in the clinical records; detailed aura subtype information was incomplete. These subgroups were reported descriptively but were not analyzed separately because the resulting sample sizes would have been small.

Age and sex data available in the clinical records were used for adjusted sensitivity analyses of the predefined ROI features. Other clinically relevant variables, including MIDAS and HIT-6 scores, sleep quality, caffeine intake, menstrual or hormonal status, and psychiatric symptom measures, were incomplete or unavailable. Multivariable adjustment was therefore limited to age and sex. None of the patients were receiving preventive headache treatment at EEG acquisition, and recordings were obtained at least 24 h after the most recent acute analgesic use and headache attack.

### 2.8. Restricted Hypothesis-Driven ROI FDR Analysis

Because the full exploratory EEG feature space included many combinations of connectivity method, frequency band, region, graph metric, and biomarker score, a restricted hypothesis-driven ROI analysis was performed to reduce the multiple-comparison burden and improve interpretability. The restricted ROI set was defined before final statistical interpretation based on biological plausibility, the prior literature on migraine and TTH network alterations, and candidate sensor-level regions expected to reflect pain-related, sensory, and temporo-parietal network involvement.

The predefined ROI hypothesis set consisted of gamma temporo-parietal coherence, gamma fronto-parietal coherence, gamma temporo-parietal wPLI, gamma temporo-parietal debiased wPLI, delta fronto-temporal imaginary coherence, gamma temporo-parietal imaginary coherence, gamma fronto-temporal imaginary coherence, and the migraine probability-like score. Benjamini–Hochberg false discovery rate correction was applied within this restricted ROI hypothesis set. Because these ROI features were evaluated within the same overall study context and no independent validation cohort was available, the findings were interpreted as internally supported candidate signatures rather than independently validated diagnostic biomarkers.

The migraine probability-like score was an exploratory composite rather than a model-derived clinical probability. It combined the prespecified migraine-oriented ROI features listed in the analysis protocol. Feature directions were aligned so that larger values consistently represented a more migraine-like pattern, after which the features were standardized and averaged. For the descriptive inferential analysis, standardization parameters were estimated from the analysis sample. Within cross-validation, all standardization and feature-processing parameters were estimated exclusively from each training fold and then applied to the corresponding validation fold. The score was treated only as a continuous summary feature; it was not calibrated as an absolute disease probability, and no diagnostic threshold or clinical-risk interpretation was assigned.

### 2.9. Statistical Analysis

Statistical analyses were performed using participant-level EEG connectivity and graph-theoretical features. Group comparisons were performed using the Kruskal–Wallis test because several EEG connectivity features were not normally distributed. For each predefined ROI feature, group-specific sample size, mean, standard deviation, median, Kruskal–Wallis H statistic, *p* value, false discovery rate-adjusted *p* value, and epsilon-squared effect size were reported.

Key pairwise contrasts were summarized using mean differences with bootstrap 95% confidence intervals and Cohen’s d with bootstrap 95% confidence intervals. ROC-AUC analyses were used to evaluate the single-feature discriminative performance of selected EEG connectivity features. Sensitivity, specificity, and AUC values were reported for exploratory interpretation.

Exploratory machine-learning analyses were performed using nested cross-validation to reduce optimistic bias. Logistic regression, LASSO logistic regression, support vector machine with radial basis function kernel, random forest, and XGBoost models were evaluated. Feature scaling, feature selection where applicable, hyperparameter tuning, and model training were performed within the cross-validation procedure to reduce data leakage. Model performance was summarized using accuracy, balanced accuracy, macro F1 score, and macro ROC-AUC.

For transparent performance reporting, accuracy, balanced accuracy, macro precision, macro recall, macro F1-score, and macro ROC-AUC were designated as evaluation metrics, with fold-wise predictions used where available to derive confusion matrices and uncertainty summaries. Feature importance was interpreted only for models with an intrinsic or permutation-based importance measure. Calibration was considered exploratory because the modest sample size and absence of an external test cohort limit stable probability calibration. Complete confusion matrices, calibration curves, and bootstrap AUC confidence intervals could not be reconstructed for every model from the retained aggregate output and are therefore identified as priorities for the fully reproducible analysis package and external-validation study; no unreported metric was inferred or imputed.

Because the machine-learning analyses were exploratory and no independent validation cohort was available, model outputs were interpreted as supportive evidence rather than clinically validated diagnostic performance.

### 2.10. Data, Code, and Protocol Availability

The anonymized data supporting the findings of this study, including derived EEG connectivity features and analysis outputs, will be made available by the corresponding author upon reasonable request, subject to institutional, ethical, and privacy restrictions. Raw clinical EEG recordings cannot be made publicly available because they were obtained in a routine clinical setting and may contain sensitive health-related information. Analysis scripts, feature definitions, and protocol details used for EEG preprocessing, connectivity estimation, statistical analysis, and machine-learning evaluation will be made available upon reasonable request to allow verification and methodological replication.

No large public database accession number is applicable to this study because the dataset was not deposited in a public repository at the time of submission.

### 2.11. Use of Generative Artificial Intelligence

Generative artificial intelligence tools were used only for language editing, grammar improvement, formatting support, and improving the clarity of the manuscript text. GenAI was not used to generate original data, create EEG recordings, perform clinical diagnosis, design the study, conduct statistical analyses, interpret the scientific results, or generate primary figures. All scientific content, analyses, interpretations, and conclusions were reviewed and approved by the authors.

## 3. Results

### 3.1. Study Population and Clinical Characteristics

A total of 150 participants were included in the EEG connectivity analysis, comprising 61 patients with migraine, 47 patients with tension-type headache (TTH), and 42 healthy controls. The available clinical and demographic characteristics are summarized in Table 1. Age and sex distributions did not differ significantly among the groups, indicating that the main diagnostic groups were broadly comparable in terms of these basic demographic variables.

The mean age was 30.35 ± 8.99 years in healthy controls, 32.46 ± 10.81 years in the migraine group, and 29.53 ± 9.84 years in the TTH group. Age did not differ significantly among the groups (*p* = 0.357). Similarly, sex distribution was not significantly different among the groups (*p* = 0.682). In this cohort, reported visual analog scale (VAS) scores were higher in the migraine group than in the TTH group (8.03 ± 1.33 vs. 7.11 ± 1.40; *p* = 0.001). Monthly headache days were 8.85 ± 5.88 in the migraine group and 10.38 ± 6.18 in the TTH group. Headache history length was 44.79 ± 72.95 months in the migraine group and 27.81 ± 31.72 months in the TTH group. Monthly headache days and headache history did not differ significantly between the headache groups. Within the migraine group, 47 patients were classified as having episodic migraine and 14 as having chronic migraine. In the TTH group, 33 patients had episodic TTH and 14 had chronic TTH. Aura status was available for only a subset of migraine records and was recorded as with or without aura; detailed aura subtypes were not consistently available. The present study was designed primarily to compare the three main diagnostic groups, and aura-based and episodic-versus-chronic subgroup analyses were not performed. These classifications are reported descriptively and should be examined in dedicated subgroup studies.

### 3.2. Global Frequency- and Method-Dependent Connectivity Profiles

Connectivity profiles depended on both frequency band and estimator. Conventional coherence was generally higher in the headache groups, particularly in TTH, whereas imaginary coherence tended to be higher in controls. The contrast between these measures suggests that the groups did not differ through a single global shift in synchronization; rather, zero-lag-sensitive and lag-sensitive coupling captured different aspects of the data.

**Figure 2:**
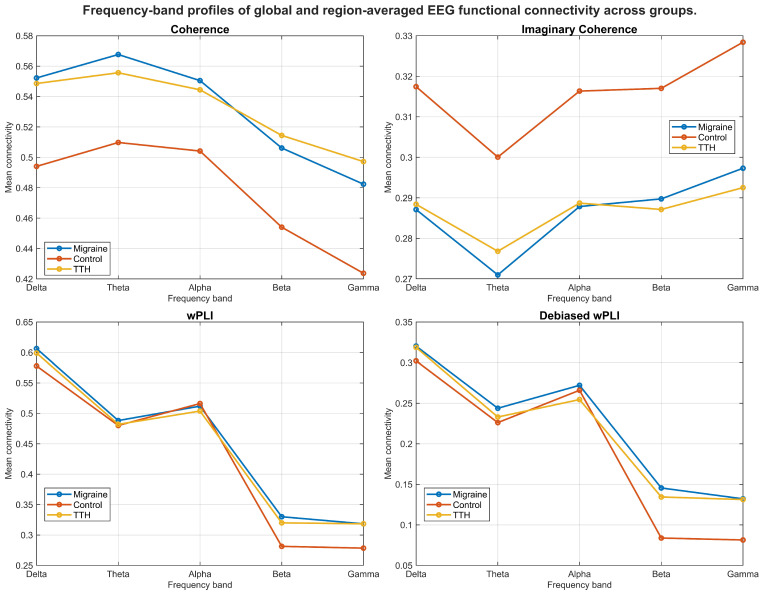
Frequency-band profiles of global and region-averaged EEG functional connectivity across groups.

Group separation in wPLI and debiased wPLI was more apparent in the beta and gamma bands than at lower frequencies. Figure 2 summarizes these frequency-dependent patterns and illustrates why the findings are best interpreted at the level of specific method–frequency combinations.

### 3.3. Group-Level Topographic Connectivity Maps

Group-level topographic maps provided spatial context for the observed connectivity differences. Coherence-based maps showed broader fronto-central, centro-parietal, temporal, and occipital increases in patients with migraine and TTH compared with healthy controls. In contrast, imaginary coherence maps showed relatively higher values in healthy controls, particularly in the posterior and fronto-temporal regions.

**Figure 3:**
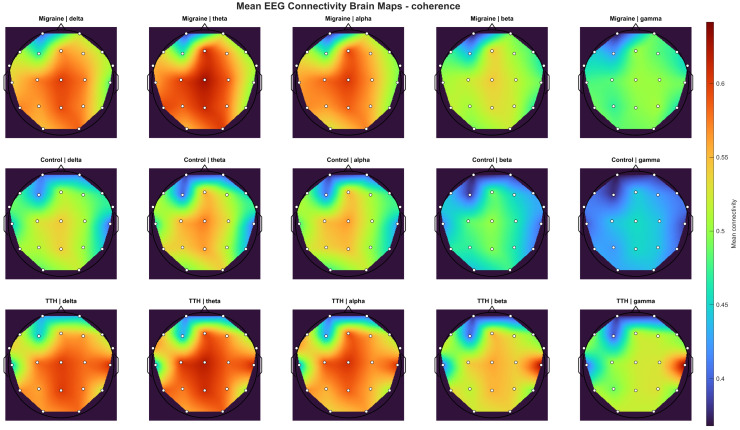
Group-level topographic maps of Coherence-Based EEG connectivity across frequency bands.

**Figure 4:**
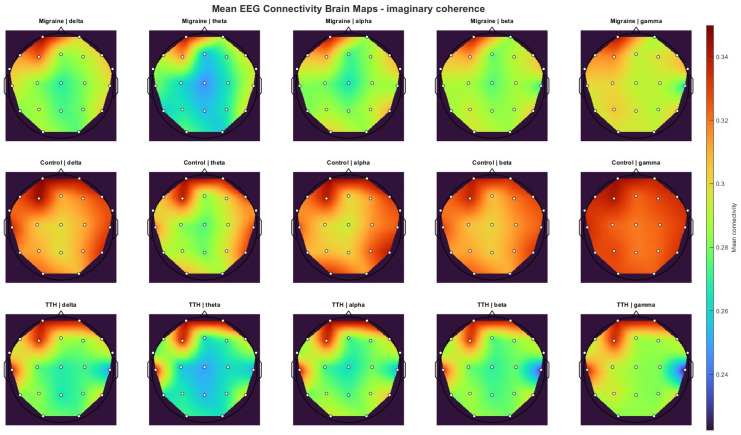
Group-level topographic maps of Imaginary Coherence-Based EEG connectivity across frequency bands.

**Figure 5:**
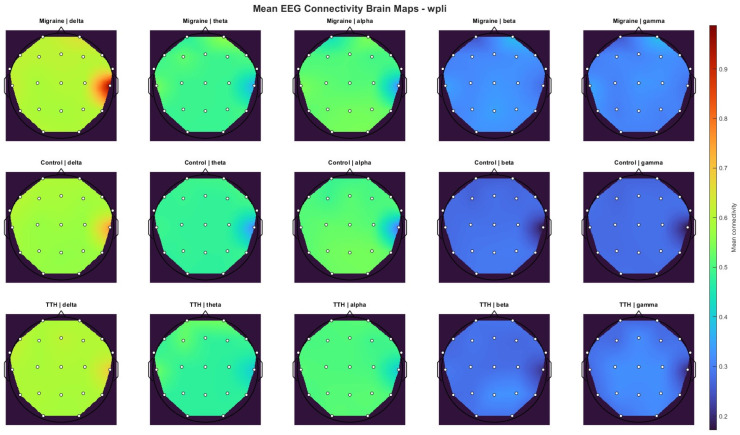
Group-level topographic maps of wPLI-based EEG connectivity across frequency bands.

**Figure 6:**
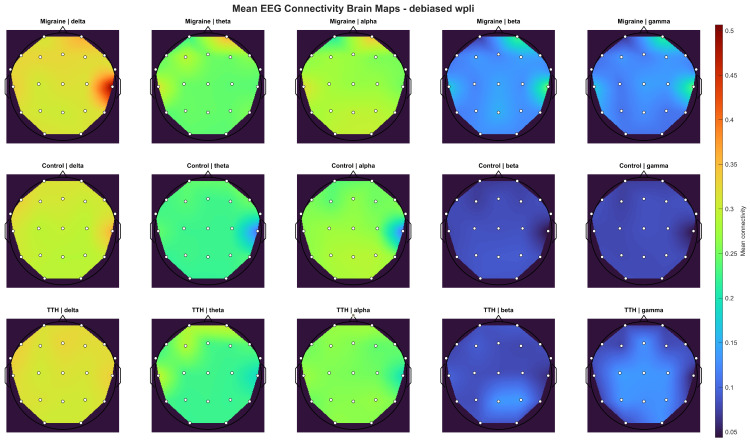
Group-level topographic maps of Debiased wPLI-based EEG connectivity across frequency bands.

The wPLI and debiased wPLI maps suggested that headache-related effects were more pronounced in beta and gamma regional patterns than in global averages. These topographic findings support the interpretation that migraine and TTH are associated with regionally specific alterations in scalp-level EEG network organization rather than a uniform global connectivity shift. Group-level topographic maps for coherence, imaginary coherence, wPLI, and debiased wPLI are shown in Figure 3, Figure 4, Figure 5 and Figure 6.

### 3.4. Restricted Hypothesis-Driven ROI FDR Analysis with Effect Sizes

Within the prespecified ROI set, all eight candidate features remained significant after false discovery rate correction (adjusted *p* = 0.0316–0.0477). Omnibus effect sizes were small to moderate. The pairwise estimates and bootstrap confidence intervals helped clarify which group contrasts accounted for these effects.

TTH showed higher gamma temporo-parietal coherence than controls (mean difference, 0.083; Cohen’s d, 0.74). Migraine showed higher gamma fronto-parietal coherence and higher gamma temporo-parietal wPLI and debiased wPLI than controls. Thus, both conventional and phase-lag-sensitive gamma-band measures contributed to separation, although their physiological meaning is not identical.

Controls, by contrast, had higher delta fronto-temporal and gamma-band imaginary coherence than the migraine group. Taken together, the results indicate a mixed pattern: selected coherence and phase-lag index measures were elevated in headache groups, while some imaginary-coherence features were reduced. Table 2 presents the full restricted ROI results.

### 3.5. Age- and Sex-Adjusted Sensitivity Analysis

Subject-level clinical matching using normalized names yielded 150 EEG records with available age and sex metadata, including 61 patients with migraine, 47 patients with TTH, and 42 healthy controls. Age- and sex-adjusted linear-model sensitivity analyses were therefore performed in this matched dataset.

Delta fronto-temporal imaginary coherence remained significantly associated with diagnostic group after adjustment for age and sex (adjusted *p* = 0.017). Other ROI effects retained their descriptive direction but were attenuated after adjustment. These findings suggest that the control-dominant imaginary coherence signature, particularly in the delta fronto-temporal feature, was relatively robust to basic demographic adjustment. However, because adjustment was limited to age and sex, these analyses should be interpreted as sensitivity analyses rather than fully adjusted confirmatory clinical models. The adjusted sensitivity analysis is shown in Table 3.

### 3.6. Topographic Difference Maps

Topographic difference maps supported the spatial interpretation of the restricted ROI findings. Coherence-based difference maps showed positive differences favoring migraine or TTH over controls mainly in central, parietal, temporal, and occipital regions. Imaginary coherence difference maps showed the opposite tendency, with healthy controls generally showing higher lag-sensitive connectivity than the headache groups.

**Figure 7:**
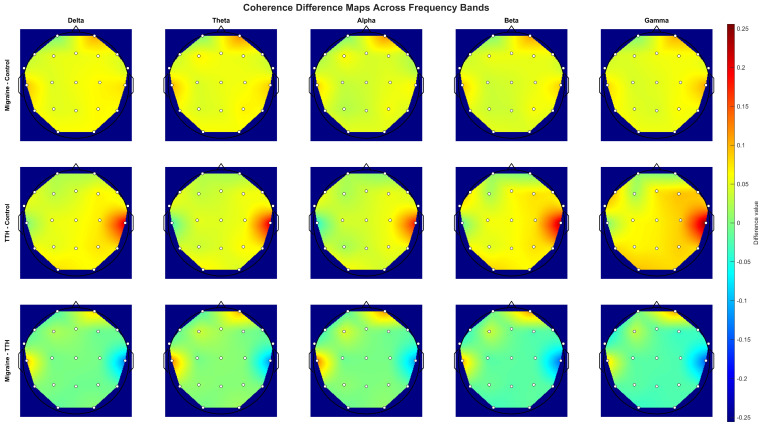
Coherence-Based topographic difference maps across EEG frequency bands.

**Figure 8:**
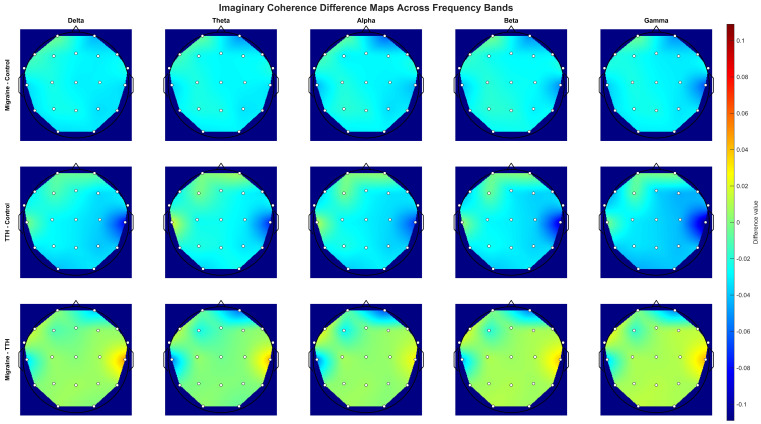
Imaginary Coherence-Based topographic difference maps across EEG frequency bands.

**Figure 9:**
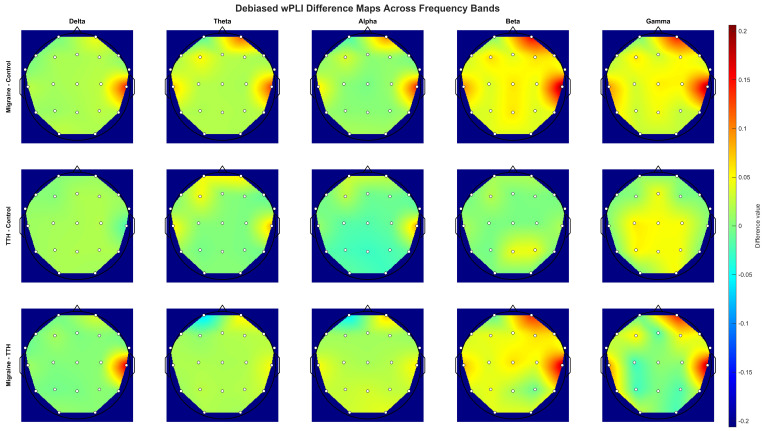
Debiased wPLI-based topographic difference maps across EEG frequency bands.

**Figure 10:**
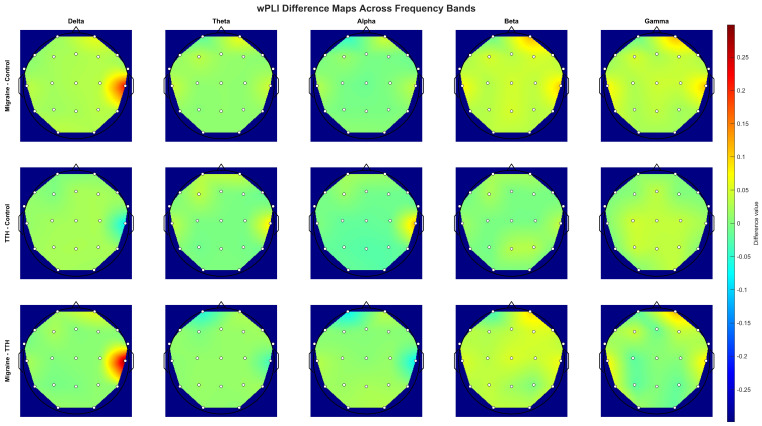
wPLI-Based Topographic Difference maps across EEG frequency bands.

The wPLI and debiased wPLI difference maps showed headache-related tendencies in the beta and gamma bands, consistent with the significant temporo-parietal phase-lag ROI results. Together, these maps indicate that the most relevant group differences were not restricted to a single electrode pair but were expressed as regional scalp-level network patterns. Topographic difference maps are shown in Figure 7, Figure 8, Figure 9 and Figure 10.

### 3.7. ROC-AUC and Machine-Learning Results

Selected single features provided moderate, but not high, discrimination. Delta fronto-temporal imaginary coherence yielded an AUC of 0.752 for migraine versus controls and 0.750 for migraine versus TTH. Gamma fronto-temporal imaginary coherence reached an AUC of 0.743 for migraine versus TTH. For TTH versus controls, gamma temporo-parietal debiased wPLI produced an AUC of 0.713, while gamma temporo-parietal coherence yielded an AUC of 0.700 for headache versus controls.

These AUC values indicate that the features contain some group-related information, but they do not establish diagnostic validity. The estimates come from a single cohort and were not tested in an independent sample. Table 4 therefore reports them as exploratory discrimination results.

The multivariable models did not materially improve three-class separation. The best balanced accuracy was 0.410 with random forest; logistic regression produced a balanced accuracy of 0.404 and a macro ROC-AUC of 0.595.

Binary headache-versus-control classification was somewhat better. XGBoost achieved 0.680 accuracy and a macro ROC-AUC of 0.653, but balanced accuracy was only 0.554. This gap shows that overall accuracy alone would overstate performance. The models should therefore be viewed as exploratory and not as clinically usable classifiers.

The modest balanced accuracy and macro-AUC values are consistent with substantial overlap among the groups. Complete model-specific confusion matrices, calibration plots, and AUC confidence intervals could not be reconstructed from the retained aggregate outputs without rerunning the fold-level pipeline. We have stated this limitation explicitly and identify out-of-fold prediction storage and complete uncertainty reporting as priorities for the reproducibility package and future external validation.

### 3.8. Summary of Main Experimental Findings

The main quantitative findings are summarized in Table 2, Table 3, Table 4 and Table 5. Restricted ROI-FDR analysis identified significant group differences in gamma temporo-parietal coherence, gamma fronto-parietal coherence, gamma temporo-parietal wPLI and debiased wPLI, delta and gamma imaginary coherence features, and the migraine probability-like score. Conventional coherence- and wPLI-based gamma features tended to be higher in headache groups, whereas imaginary coherence features were generally higher in healthy controls.

Single-feature ROC-AUC analyses showed moderate discriminative performance for selected EEG connectivity features, particularly delta fronto-temporal imaginary coherence and gamma temporo-parietal debiased wPLI. However, nested cross-validated machine-learning models showed modest classification performance, especially for three-class discrimination. Overall, the results support the presence of sensor-level EEG connectivity alterations in migraine and TTH, but they should be interpreted as exploratory candidate signatures rather than clinically validated diagnostic biomarkers.

## 4. Discussion

This study investigated resting-state EEG functional connectivity and sensor-level network organization in patients with migraine, patients with tension-type headache (TTH), and healthy controls using a multimethod analytical framework. By combining coherence, imaginary coherence, wPLI, debiased wPLI, topographic mapping, graph-theoretical features, ROC-AUC analysis, and nested cross-validated machine learning, the study aimed to determine whether primary headache disorders show distinct EEG connectivity signatures rather than a single uniform abnormality. The main finding was that the restricted ROI-FDR analysis identified significant method-dependent and region-specific connectivity differences among the three groups. TTH was characterized by higher gamma temporo-parietal coherence, migraine by higher gamma temporo-parietal wPLI/debiased wPLI and a higher migraine probability-like score, and healthy controls by higher fronto-temporal and temporo-parietal imaginary coherence.

These findings are consistent with the multidimensional view of primary headache disorders emphasized in previous studies. Both migraine and TTH have been associated with alterations in pain processing and sensory-network function, although the underlying mechanisms and the strength of evidence differ between the disorders [2,3,5,6]. In TTH, peripheral musculoskeletal mechanisms, craniocervical dysfunction, myofascial factors, psychosocial stressors, and central pain modulation may interact and contribute to clinical heterogeneity, with central sensitization being particularly relevant in chronic TTH [6,16]. In migraine, abnormal cortical responsiveness, sensory hypersensitivity, trigeminovascular mechanisms, and altered brain network dynamics have frequently been reported [3,4,5,10,11,15]. The present EEG findings support this broader framework by suggesting that migraine and TTH are associated with different sensor-level network patterns rather than identical connectivity abnormalities.

TTH showed the highest gamma temporo-parietal coherence. Because coherence reflects conventional linear synchronization and is sensitive to both physiological coupling and zero-lag components, this finding indicates a broader sensor-level synchronization pattern in TTH. It may be compatible with altered integration of somatosensory, attentional, and pain-related information, but this interpretation remains tentative. Recent fMRI and MEG studies have reported abnormal functional connectivity and disrupted network topology in TTH involving frontal, temporal, somatomotor, thalamic, insular, and visual-related systems [7,8,9]. This finding is also compatible with a multidimensional model of TTH in which peripheral and central mechanisms may interact dynamically [6]. However, because coherence is sensitive to volume conduction, common-source effects, and residual cranial muscle activity, this result should be interpreted as a sensor-level synchronization pattern rather than direct evidence of increased cortical source-level communication.

Migraine showed a different connectivity profile. Gamma temporo-parietal wPLI and debiased wPLI were highest in migraine and remained significant after FDR correction within the restricted ROI hypothesis set. Unlike conventional coherence, wPLI and debiased wPLI emphasize non-zero phase-lag relationships and reduce the influence of zero-lag coupling. Therefore, the migraine-related increase in gamma temporo-parietal phase-lag-sensitive connectivity indicates stronger non-zero-lag sensor-level coupling in the measured connections, but its functional significance remains uncertain. Temporo-parietal and posterior cortical systems are involved in multisensory integration, attentional modulation, visual-somatosensory processing, and pain-related dynamics; however, the present sensor-level findings do not establish a specific cortical mechanism. Previous EEG and MEG studies have reported altered coherence, microstate dynamics, oscillatory activity, and functional connectivity in migraine [15]. Because low-gamma sensor-level activity may be affected by residual cranial muscle activity, these findings cannot be attributed exclusively to cortical gamma synchronization.

Imaginary coherence showed an opposite, control-dominant pattern. Delta fronto-temporal imaginary coherence and gamma fronto-temporal/temporo-parietal imaginary coherence were higher in healthy controls than in headache groups. Imaginary coherence is less sensitive to zero-lag coupling and is commonly interpreted as a lag-sensitive connectivity measure [16]. The higher imaginary coherence observed in controls indicates stronger non-zero-lag sensor-level coupling in the measured connections; its functional significance remains uncertain. Therefore, higher conventional coherence in headache groups should not be interpreted simply as more efficient physiological communication, and lower imaginary coherence should not be assumed to represent impaired communication without independent physiological validation.

A plausible explanation for the apparent divergence between increased gamma coherence and reduced imaginary coherence is that the estimators emphasize different signal components. Conventional coherence includes near-zero-lag synchronization and can increase because of common inputs, reference effects, field spread, residual muscle activity, or genuinely more synchronous local activity. Imaginary coherence suppresses the zero-lag component and preferentially reflects delayed interactions. Thus, the combination observed here may reflect stronger shared or locally synchronized gamma activity together with lower non-zero-lag coupling, rather than a simple global increase or decrease in physiological connectivity. Prior migraine studies have reported heterogeneous coherence and phase-lag findings across frequency bands, recording states, treatments, montages, and preprocessing pipelines [3,4,5,10,11,12,13,15]. Differences from earlier reports may therefore arise from the operational timing of post-attack recordings, clinical heterogeneity, 21-channel spatial resolution, artifact handling, reference choice, ROI construction, and the use of distinct connectivity estimators.

Methodological choices can materially influence resting-state EEG connectivity estimates. Differences in referencing, artifact handling, epoch selection, spectral estimation, and connectivity metrics may change both effect magnitude and direction. The present findings should therefore be interpreted in relation to the specific preprocessing and estimation pipeline used in this study [16].

Although imaginary coherence, wPLI, and debiased wPLI are less sensitive to zero-lag coupling than conventional coherence, they do not eliminate volume conduction or field spread. The temporo-parietal and fronto-temporal results therefore describe connectivity among scalp electrodes, not direct interactions between cortical sources. High-density EEG with source reconstruction, beamforming, or multimodal EEG-fMRI will be needed to test the anatomical specificity of these patterns [18].

The effect-size estimates quantified the magnitude of the main between-group differences beyond statistical significance alone. The largest reported contrast was the higher gamma temporo-parietal coherence in TTH than in controls, while several migraine-control contrasts were smaller and had wider confidence intervals. These estimates support the statistical pattern but do not, by themselves, establish biological or clinical importance.

Clinical and demographic findings provide additional context for the EEG results. Age and sex distributions were broadly comparable across groups, and the delta fronto-temporal imaginary-coherence effect remained significant after adjustment for these variables. Adjustment for age and sex alone, however, cannot exclude residual confounding.

The unavailable covariates could have influenced both oscillatory activity and connectivity estimates. Acute and preventive medications may alter cortical excitability; sleep loss and circadian disruption can change spectral power and network coupling; caffeine can modify arousal-related rhythms; aura status may define a neurophysiologically distinct migraine subgroup; hormonal phase may affect excitability and attack susceptibility; and anxiety or depressive symptoms may alter pain, attention, and fronto-limbic dynamics. Residual confounding is therefore possible even where age- and sex-adjusted effects remained directionally consistent. Future studies should prospectively collect these variables using standardized instruments and incorporate them into prespecified multivariable or hierarchical models, including interactions where biologically justified and sensitivity analyses stratified by aura, medication exposure, sleep quality, and hormonal status.

Several individual features showed moderate ROC-AUC values, particularly delta fronto-temporal imaginary coherence. This indicates measurable between-group discrimination, but the observed performance remains below the level required for clinical use. The estimates were derived from a single cohort and should be confirmed in an independent sample.

The machine-learning analyses similarly showed limited discriminatory performance. Three-class classification was weak, and the binary headache-versus-control models achieved only modest balanced accuracy despite higher overall accuracy. Balanced accuracy therefore provided a more conservative and informative estimate of performance than overall accuracy. These results are consistent with diagnostic overlap, the limited sample size, and the use of leakage-conscious validation.

Graph-theoretical findings should also be interpreted cautiously. Although graph metrics provide useful summary measures of network organization, their stability depends on thresholding strategy, network density, channel montage, connectivity estimator, and preprocessing decisions. In the present dataset, graph-theoretical measures did not outperform hypothesis-driven regional connectivity features or candidate biomarker scores. This may reflect the limited spatial resolution of the 21-channel montage, the sensitivity of graph metrics to sparse-network construction, and the modest separation of whole-network properties among the three groups. Therefore, the graph results should be considered exploratory. Future graph analyses may benefit from high-density EEG, source-level reconstruction, standardized network-density ranges, threshold-sensitivity analysis, and harmonized preprocessing pipelines.

Across the ROI, topographic, effect-size, ROC, and sensitivity analyses, two themes were consistent: temporo-parietal gamma connectivity differed across headache groups, and fronto-temporal imaginary coherence was relatively higher in controls. This convergence supports further study of these signals. It does not, however, establish a clinically reliable biomarker. External replication, complete covariate assessment, source-level confirmation, and prespecified validation are still required.

The present findings do not support diagnosis, screening, or treatment selection at the individual-patient level. After external validation, a connectivity panel might instead be evaluated as an adjunct for neurophysiological subgrouping, longitudinal monitoring, or treatment-response research. Clinical translation would require standardized acquisition and preprocessing, normative reference data, prespecified thresholds, calibration and decision-curve analyses, and multicenter evidence that EEG adds information beyond routine clinical assessment.

Resting-state EEG connectivity should not be considered a stand-alone diagnostic test for migraine or TTH on the basis of these data. Its more realistic future role may be to complement clinical evaluation by characterizing network-level heterogeneity. That possibility remains hypothetical until the findings are replicated prospectively and tested against established clinical predictors.

### Limitations and Future Directions

This study has several limitations. It was conducted in a single cohort without external validation, so the identified features are candidate signatures rather than established biomarkers. Several clinically relevant covariates were incomplete, including MIDAS, HIT-6, detailed aura information, acute and preventive medication exposure, sleep quality, caffeine intake, menstrual or hormonal status, psychiatric symptoms, and time since the last attack. Adjustment was therefore limited to age and sex, and residual confounding remains possible. Because migraine postdromal symptoms may persist for up to 48 h, the required 24 h interval after headache resolution may not have completely excluded early post-attack effects. In addition, low-gamma sensor-level activity may have been affected by residual cranial muscle activity because uniform ICA-based artifact correction was not available; gamma findings therefore cannot be attributed exclusively to cortical synchronization. Feature-specific quality-control filtering also reduced the valid sample size for several ROI measures and may have introduced selection bias. Finally, the retained aggregate machine-learning outputs did not permit complete reconstruction of fold-level confusion matrices, calibration curves, or uncertainty estimates for every model.

The 21-channel montage provided limited spatial sampling. Graph nodes represented scalp electrodes rather than reconstructed cortical sources, and neither imaginary coherence nor phase-lag indices can fully remove the effects of volume conduction and field spread. The restricted ROI analysis was hypothesis-driven but was evaluated in the same cohort as the broader exploratory analyses; its corrected findings therefore require independent confirmation. Future studies should use high-density EEG, prospectively specified preprocessing and ROI definitions, complete clinical phenotyping, stored out-of-fold predictions, and external multicenter validation.

## 5. Conclusions

This study identified frequency-, method-, and region-dependent differences in resting-state EEG connectivity among migraine, TTH, and healthy controls. TTH showed higher gamma temporo-parietal coherence, migraine showed higher gamma temporo-parietal wPLI and debiased wPLI, and controls showed higher selected imaginary-coherence features. These findings indicate that conventional and phase-lag-sensitive estimators capture different aspects of the group differences.

The agreement across ROI, topographic, effect-size, ROC, and age- and sex-adjusted analyses supports further investigation. However, the modest machine-learning performance, feature-specific data loss, sensor-level design, and absence of external validation preclude clinical use. Independent multicenter studies with denser EEG, source-level analysis, and fuller clinical covariate assessment are required.

## Figures and Tables

**Table 1:** Available clinical and demographic characteristics.

Group	Clinical Records	Age, Mean ± SD	Female/Male	VAS, Mean ± SD	Monthly Headache Days (Days), Mean ± SD	Complaint Duration (Months), Mean ± SD	Episodic/Chronic
Control	42	30.35 ± 8.99	29/13	NA	NA	NA	NA
Migraine	61	32.46 ± 10.81	45/16	8.03 ± 1.33	8.85 ± 5.88	44.79 ± 72.95	47/14
TTH	47	29.53 ± 9.84	37/10	7.11 ± 1.40	10.38 ± 6.18	27.81 ± 31.72	33/14

**Table 2:** Concise summary of restricted ROI-FDR EEG connectivity signatures. Detailed pairwise mean differences and bootstrap confidence intervals are provided in Table A1.

Candidate EEG Feature	N C/M/T	Control	Migraine	TTH	*p*	FDR-p	Epsilon^2^	Highest Group
Gamma coherence, temporo-parietal	26/43/31	0.488	0.565	0.571	0.0103	0.0316	0.074	TTH
Gamma coherence, fronto-parietal	25/43/31	0.428	0.488	0.483	0.0477	0.0477	0.043	Migraine
Gamma wPLI, temporo-parietal	26/43/31	0.275	0.338	0.317	0.0396	0.0453	0.046	Migraine
Gamma debiased wPLI, temporo-parietal	26/43/31	0.078	0.154	0.124	0.0197	0.0316	0.060	Migraine
Delta imaginary coherence, fronto-temporal	14/19/16	0.318	0.281	0.315	0.0109	0.0316	0.153	Control
Gamma imaginary coherence, temporo-parietal	26/43/31	0.305	0.269	0.269	0.0142	0.0316	0.067	Control
Gamma imaginary coherence, fronto-temporal	14/19/16	0.323	0.280	0.312	0.0196	0.0316	0.127	Control
Migraine probability-like score	26/43/31	0.382	0.479	0.452	0.0391	0.0453	0.046	Migraine

Note: The N C/M/T column indicates the number of valid ROI-level observations included for each feature after feature-specific quality-control filtering, not the total number of participants in each diagnostic group. The full EEG sample consisted of 42 healthy controls, 61 patients with migraine, and 47 patients with TTH. FDR correction was applied within the predefined restricted ROI hypothesis set. Epsilon-squared represents the Kruskal–Wallis omnibus effect size. Mean-difference and Cohen’s d confidence intervals were estimated by bootstrap resampling for the key contrast selected according to the hypothesized direction.

**Table 3:** Age- and sex-adjusted sensitivity analysis in the full clinically matched EEG dataset.

ROI Feature	Age- and Sex-Adjusted Group (*p*)	Interpretation
Gamma coherence, temporo-parietal	0.057	Directionally consistent/attenuated
Gamma coherence, fronto-parietal	0.585	Directionally consistent/attenuated
Gamma wPLI, temporo-parietal	0.497	Directionally consistent/attenuated
Gamma debiased wPLI, temporo-parietal	0.539	Directionally consistent/attenuated
Delta imaginary coherence, fronto-temporal	0.017	Significant
Gamma imaginary coherence, temporo-parietal	0.146	Directionally consistent/attenuated
Gamma imaginary coherence, fronto-temporal	0.102	Directionally consistent/attenuated
Migraine probability-like score	0.417	Directionally consistent/attenuated

Note: Age and sex metadata were available for all 150 EEG records. Age- and sex-adjusted models were performed as sensitivity analyses and should not be interpreted as fully powered confirmatory models.

**Table 4:** Exploratory ROC-AUC performance of selected EEG connectivity features.

Comparison	Best Candidate Feature	AUC	Sensitivity	Specificity	Interpretation
Migraine vs. control	Delta imaginary coherence, fronto-temporal	0.752	0.579	1.000	Moderate
Migraine vs. TTH	Delta imaginary coherence, fronto-temporal	0.750	0.842	0.688	Moderate
Migraine vs. TTH	Gamma imaginary coherence, fronto-temporal	0.743	0.684	0.813	Moderate
TTH vs. control	Gamma debiased wPLI, temporo-parietal	0.713	0.581	0.808	Moderate
Headache vs. control	Gamma coherence, temporo-parietal	0.700	0.527	0.846	Moderate
Migraine vs. control	Migraine probability-like score	0.689	0.628	0.769	Exploratory composite

**Table 5:** Nested cross-validated machine-learning performance based on the retained aggregate outputs.

Task	Best Model	Accuracy	Balanced Accuracy	Macro Precision/Recall/F1	Macro ROC-AUC	Interpretation
Three-class	Random forest	NR	0.410	NR	NR	Weak discrimination
Three-class	Logistic regression	NR	0.404	NR	0.595	Weak discrimination
Headache vs. control	XGBoost	0.680	0.554	NR	0.653	Modest; not clinically usable

Note: NR, not reconstructable from the retained aggregate output. No unreported metric was inferred or imputed.

## Data Availability

The data supporting the findings of this study are available from the corresponding author upon reasonable request, subject to institutional, ethical, and privacy restrictions. Raw EEG recordings cannot be made publicly available because they were obtained in a routine clinical setting and may contain sensitive health-related information. Derived anonymized feature tables and analysis outputs may be shared upon reasonable request where permitted by the relevant ethical and institutional regulations.

## Abbreviations

EEG	Electroencephalography
TTH	Tension-type headache
ROI	Region of interest
FDR	False discovery rate
wPLI	Weighted phase-lag index
AUC	Area under the curve
ROC	Receiver operating characteristic
VAS	Visual analog scale
MIDAS	Migraine Disability Assessment
HIT-6	Headache Impact Test-6
ICA	Independent component analysis
SVM-RBF	Support vector machine with radial basis function kernel
LASSO	Least absolute shrinkage and selection operator
XGBoost	Extreme gradient boosting
CI	Confidence interval
SD	Standard deviation
eLORETA	Exact low-resolution electromagnetic tomography
sLORETA	Standardized low-resolution electromagnetic tomography

## Appendix A

**Table A1:** Complete restricted ROI-FDR results, including key contrasts, mean differences, Cohen’s d values, and bootstrap 95% confidence intervals.

Candidate EEG Feature	N C/M/T	Control	Migraine	TTH	*p*	FDR-p	Epsilon^2^	Highest Group	Key Contrast	Mean Difference (95% CI)	Cohen’s d (95% CI)
Gamma coherence, temporo-parietal	26/43/31	0.488	0.565	0.571	0.0103	0.0316	0.074	TTH	TTH vs. Control	0.083 (0.032 to 0.141)	0.74 (0.37 to 1.12)
Gamma coherence, fronto-parietal	25/43/31	0.428	0.488	0.483	0.0477	0.0477	0.043	Migraine	Migraine vs. Control	0.060 (0.016 to 0.110)	0.49 (0.24 to 0.72)
Gamma wPLI, temporo-parietal	26/43/31	0.275	0.338	0.317	0.0396	0.0453	0.046	Migraine	Migraine vs. Control	0.063 (0.015 to 0.129)	0.42 (0.23 to 0.63)
Gamma debiased wPLI, temporo-parietal	26/43/31	0.078	0.154	0.124	0.0197	0.0316	0.060	Migraine	Migraine vs. Control	0.075 (0.013 to 0.159)	0.41 (0.25 to 0.63)
Delta imaginary coherence, fronto-temporal	14/19/16	0.318	0.281	0.315	0.0109	0.0316	0.153	Control	Control vs. Migraine	0.037 (0.010 to 0.063)	0.84 (0.27 to 1.46)
Gamma imaginary coherence, temporo-parietal	26/43/31	0.305	0.269	0.269	0.0142	0.0316	0.067	Control	Control vs. Migraine	0.036 (0.013 to 0.061)	0.57 (0.34 to 0.81)
Gamma imaginary coherence, fronto-temporal	14/19/16	0.323	0.280	0.312	0.0196	0.0316	0.127	Control	Control vs. Migraine	0.043 (0.012 to 0.079)	0.74 (0.31 to 1.28)
Migraine probability-like score	26/43/31	0.382	0.479	0.452	0.0391	0.0453	0.046	Migraine	Migraine vs. Control	0.097 (0.036 to 0.166)	0.64 (0.26 to 1.04)

**Table A2:** Sensor-Level Electrode-Pair Definitions for Regional EEG Connectivity Analysis.

ROI	Electrode Pairs
Fronto-central	F3-C3; F4-C4; Fz-Cz; F3-Cz; F4-Cz
Fronto-parietal	F3-P3; F4-P4; Fz-Pz; F3-Pz; F4-Pz
Fronto-temporal	F7-T3; F8-T4; F3-T3; F4-T4
Temporo-parietal	T3-P3; T4-P4; T5-P3; T6-P4
Parieto-occipital	P3-O1; P4-O2; Pz-O1; Pz-O2; O1-O2
Interhemispheric	Fp1-Fp2; F3-F4; C3-C4; P3-P4; O1-O2; T3-T4; T5-T6
